# Synthesis of Site-Specific Antibody–[60]Fullerene–Oligonucleotide Conjugates for Cellular Targeting

**DOI:** 10.1021/acsabm.3c00318

**Published:** 2023-07-11

**Authors:** Antti Äärelä, Kati Räsänen, Patrik Holm, Harri Salo, Pasi Virta

**Affiliations:** † Department of Chemistry, University of Turku, FI-20500 Turku, Finland; ‡ Research and Development, Orion Pharma, FI-20380 Turku, Finland

**Keywords:** Molecular Spherical Nucleic Acid, Antibody−Oligonucleotide Conjugate, [60]Fullerene Conjugate, Nanoparticle, Targeted Delivery

## Abstract

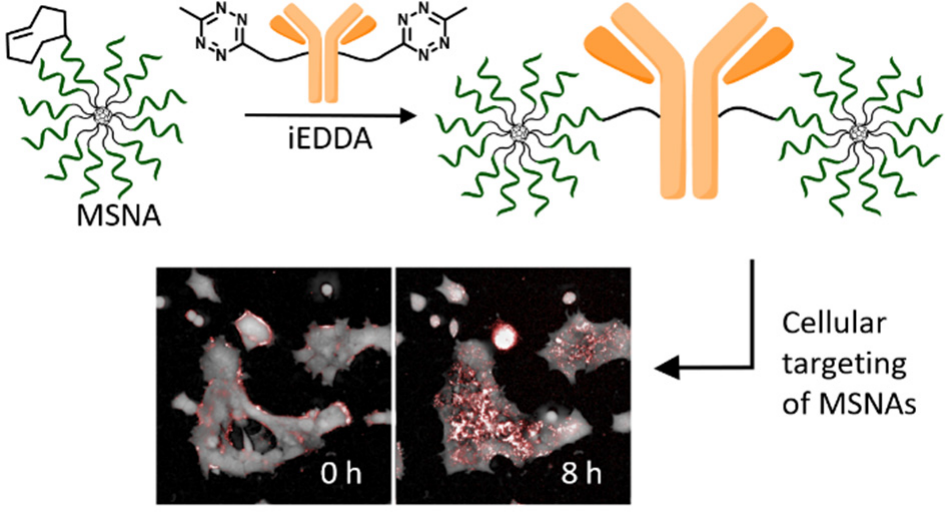

An ideal therapeutic antibody–oligonucleotide conjugate (AOC) would be a uniform construct, contain a maximal oligonucleotide (ON) payload, and retain the antibody (Ab)-mediated binding properties, which leads to an efficient delivery of the ON cargo to the site of therapeutic action. Herein, [60]fullerene-based molecular spherical nucleic acids (MSNAs) have been site-specifically conjugated to antibodies (Abs), and the Ab-mediated cellular targeting of the MSNA–Ab conjugates has been studied. A well-established glycan engineering technology and robust orthogonal click chemistries yielded the desired uniform MSNA–Ab conjugates (MW ∼ 270 kDa), with an oligonucleotide (ON):Ab ratio of 24:1, in 20–26% isolated yields. These AOCs retained the antigen binding properties (Trastuzumab’s binding to human epidermal growth factor receptor 2, HER2), studied by biolayer interferometry. In addition, Ab-mediated endocytosis was demonstrated with live-cell fluorescence and phase-contrast microscopy on BT-474 breast carcinoma cells, overexpressing HER2. The effect on cell proliferation was analyzed by label-free live-cell time-lapse imaging.

## Introduction

Antibody–oligonucleotide conjugates (AOCs) offer a versatile tool for diagnostic and therapeutic applications.^1^ The antibody (Ab) constituent of these chimeric bioconjugates acts as a target-recognizing element, and the oligonucleotide (ON) one serves as a therapeutic agent or a reporter group. During recent years the development of therapeutic AOCs has accelerated and also entered clinical trials.^2^ In addition to therapeutics, AOCs can be used as diagnostic agents, e.g., in immuno-PCR,^3−6^ various proximity assays,^7−12^ and protein arrays.^13^ Covalent Ab conjugation utilizes typically reactive Lys,^14^ Cys,^15^ Tyr,^16^ and Arg^17^ residues. These approaches produce conjugates that are heterogeneous regarding conjugation sites and degree of labeling. Unspecific conjugation may also lead to poor reproducibility and complicated analytics due to the promiscuous conjugation.^18,19^ The ideal therapeutic AOC would be a uniform construct, retain the Ab-mediated binding/delivery properties, and contain a maximal ON payload, which will be finally released to the site of action in the cytoplasm of target cells. The retained delivery properties may be a hard task, as the negatively charged ON payload may, via electrostatic interaction, mask the active binding sites or alter the native conformation of the Ab constituent. Successful targeted delivery has been demonstrated with AOCs of an average Ab:ON ratio of 1:6.^20^

Spherical nucleic acids (SNAs)^21−28^ are nanostructures consisting of an appropriate core unit and a dense layer of ONs. The dense layer of ONs can activate Scavenger A receptors that lead to an enhanced cellular uptake. This rather universal receptor-mediated endocytosis cannot distinguish healthy cells, and therefore SNAs are applicable for topical delivery primarily.^28,29^ However, depending on the particle size, multivalency of ONs, and structural design of SNAs, the radial formulation may hide negative distribution properties of ONs and offer more possibilities for the targeted delivery, when these nucleic acid derivatives are integrated with tissue-specific ligands. Conjugation of SNAs to Abs would result in AOCs of high ON payload that are applicable for cell-/tissue-specific gene regulation. A noncovalent conjugate of a gold nanoparticle-based polydisperse SNA and an Ab has previously been reported.^30^ A monoclonal Ab–DNA conjugate was hybridized to an SNA, which gave an average Ab–SNA ratio of 2:1. These polydisperse macromolecular hybrid constructs exhibited cell type selectivity with an enhanced uptake and gene knock down compared with naked SNAs.

**Scheme 1:**
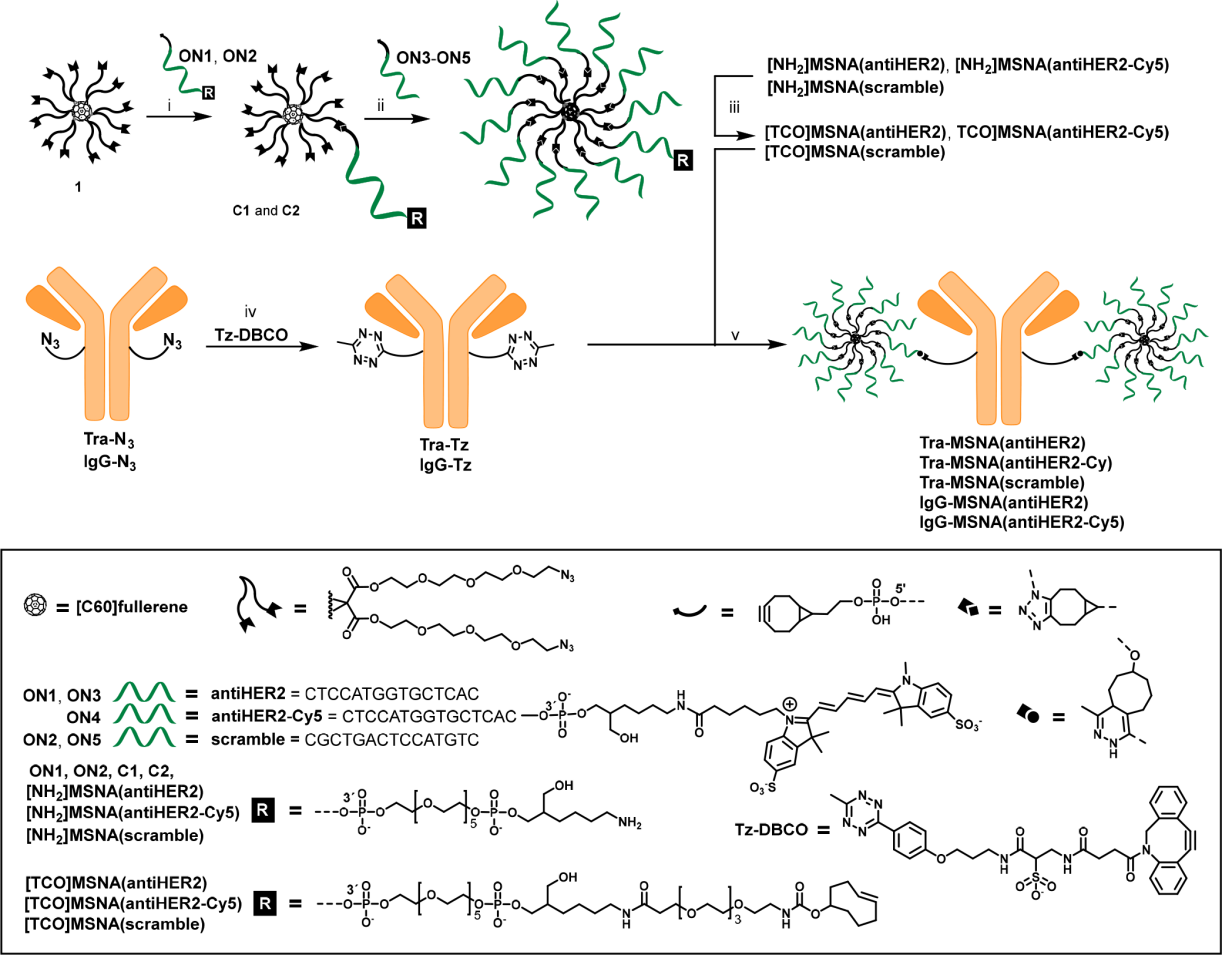
Synthesis of Ab–MSNA Conjugates Conditions: (i) BCN-modified oligonucleotide, C_60_ core **1** (4 equiv) in DMSO, overnight at rt, (ii) **C1** or **C2**, BCN-modified oligonucleotides **ON3–ON5** (1.2 equiv/arm) in aqueous 1.5 M NaCl, 3 days at rt; (iii) TCO-PEG_4_-NHS ester, 0.1 M sodium borate (pH 8.5), 4 h at rt; (iv) Tz-DBCO (100 equiv) in PBS (pH 7.4), 4 h at rt; (v) **Tz-Ab**, **[TCO]MSNA** (8 equiv) in PBS (pH 7.4), 4 h at rt.

In the present study, uniform covalent conjugates of molecular spherical nucleic acids, MSNAs, and Abs have been synthesized for the first time (Scheme 1), and the Ab-mediated cellular targeting of the conjugates has been studied. Among potential multivalent branching units, an azide-modified [C60]fullerene (**1**, Scheme 1)^27,31^ was selected for the assembly of MSNAs. Its synthetic availability via Bingel cyclopropanation,^32^ radially symmetric structure, and in particular a controlled monofunctionalization technique^33^ have made it an attractive core structure for MSNAs.^25,33^ Furthermore, a 12-arm [C60]-based MSNA, consisting of an antisense ON that targets HER2 (Human Epidermal Growth Factor Receptor 2), has been shown to present sufficient oligonucleotide density to activate the Scavenger receptor A mediated cellular uptake, resulting in enhanced downregulation of HER2.^27^ The MSNAs, which consisted of either an anti-HER2 ON sequence or the corresponding scrambled one (Scheme 1), were assembled using strain-promoted azide–alkyne cycloaddition (SPAAC)^34^ between the azide-modified [60]fullerene core **1** and bicyclononyne (BCN)-modified ONs. Our published stepwise-SPAAC method,^33^ followed by a selective amide coupling, yielded mono-*trans*-cyclooctene (TCO)-modified [60]fullerene-based MSNAs. Trastuzumab (**Tra**) and an isotype control monoclonal Ab (**IgG**) were used as Ab constituents. Tra’s (brand name Herceptin)^35^ therapeutic action is based on binding to HER2, and it is broadly used as a model in the development of antibody–drug conjugate technologies.^36^ Abs were site-specifically modified by the azide group using an established glycan engineering technology (GlyCLICK)^37,38^ and exposed then to SPAAC with a bifunctional DBCO/tetrazine linker. Inverse electron-demand Diels–Alder reaction (iEDDA)^39,40^ between the TCO-modified MSNAs and the tetrazine-modified Abs afforded the desired AOCs with an Ab-to-ON ratio of 1:24. The AOCs were purified by size-exclusion chromatography (SEC), and their authenticity was verified by reducing polyacrylamide gel electrophoresis (SDS-PAGE) and size-exclusion chromatography equipped with a multiple-angle light-scattering detector (SEC-MALS). The binding affinity of Tra–MSNA conjugates to HER2 protein was studied by biolayer interferometry. The receptor-mediated endocytosis on breast carcinoma cells (BT-474) was studied with Cyanine5-labeled conjugates. The effect of the Tra–MSNA conjugate on BT-474 cell proliferation was analyzed by label-free live-cell time-lapse imaging. In all assays the corresponding IgG–MSNA conjugate was used as a negative control to affirm that the target recognition was specific. Overall, this study demonstrates that uniform and site-specific MSNA–Ab conjugates can be synthesized in a controlled manner. Furthermore, these conjugates, despite the high ON payload (Ab–ON ratio of 1:24), can retain the antigen binding properties (binding of Tr to HER2 protein) and undergo Ab-mediated endocytosis. Such constructs may be applied to the targeted delivery of the MSNAs and in an ideal case find the synergistic effect between the Ab and ON constituents.

## Experimental Section

### Reagents

Sulfo-cyanine-5-NHS (1-[6-(2,5-dioxopyrrolidin-1-yloxy)-6-oxohexyl]-3,3-dimethyl-2-[(1*E*,3*E*,5*E*)-5-(1,3,3- trimethyl-5-sulfonatoindolin-2-ylidene)penta-1,3-dienyl]-3*H*-indolium-5-sulfonate), TCO-PEG_4_-NHS ester ((2,5-dioxopyrrolidin-1-yl) 3-[2-[2-[2-[2-[[(4*Z*)-cyclooct-4-en-1-yl]oxycarbonylamino]ethoxy]ethoxy]ethoxy]ethoxy]propanoate), and Tz-DBCO (3-[[4-(2-azatricyclo[10.4.0.04,9]hexadeca-1(16),4,6,8,12,14-hexaen-10-yn-2-yl)-4-oxobutanoyl]amino]-1-[3-[4-(6-methyl-1,2,4,5-tetrazin-3-yl)phenoxy]propylamino]-1-oxopropane-2-sulfonic acid) were purchased from Jena Biosciences (Jena, Germany). Reagents used in oligonucleotide synthesis were purchased from Glen Research (Sterling, USA), LGC Bioresearch (Teddington, UK), and Cytiva (Marlborough, USA). Other reagents were purchased from Sigma-Aldrich (St. Louis, USA). All reagents were used as received.

### Synthesis of Oligonucleotides

For the assembly of MSNAs (Scheme 1), BCN-modified ONs (**ON1**–**ON5**) were synthesized using an automated DNA/RNA synthesizer. A standard phosphoramidite coupling cycle and commercially available 2′-deoxyribonucleotide building blocks were used for the assembly. The ONs were released from the solid support/deprotected by concentrated ammonia and purified by RP-HPLC using a semipreparative column (250 × 10 mm, 5 μm), a linear gradient from 5% to 45% MeCN in 50 mmol L^–1^ of triethylammonium acetate over 25 min, a flow rate of 3.0 mL min^–1^, and detection at 260 nm. Cyanine5-labeled **ON4** was synthesized by treating the corresponding 3′-amino- and 5′-BCN-modified ON (0.8 μmol in 2 mL of 0.1 M sodium borate, pH 8.5) with an activated succinimidyl ester of sulfo-cyanine 5 (1-[6-(2,5-dioxopyrrolidin-1-yloxy)-6-oxohexyl]-3,3-dimethyl-2-[(1*E*,3*E*,5*E*)-5-(1,3,3-trimethyl-5-sulfonatoindolin-2-ylidene)penta-1,3-dienyl]-3*H*-indolium-5-sulfonate, 7.5 μmol in 200 μL of DMSO). The reaction mixture was gently shaken for 4 h at rt and subjected to RP-HPLC for purification (25% isolated yield for the labeling). The authenticity of the oligonucleotides was verified by MS (ESI-TOF) (electrospray ionization time-of-flight) (Table S1).

### Synthesis of C60–ON Conjugates **C1** and **C2**

BCN-modified oligonucleotides **ON1** or **ON2** (0.2 μmol in 100 μL of H_2_O) were added to a mixture of [60]fullerene core **1**(27,32) (0.8 μmol in 900 μL of DMSO) in a microcentrifuge tube, and the reaction mixture was shaken gently overnight at room temperature. The reaction was purified by RP-HPLC using a semipreparative column (250 × 10 mm, 5 μm), a gradient elution from 40% to 100% MeCN in 50 mmol L^–1^ of triethylammonium acetate over 30 min, and detection at 260 nm. The product fractions were collected and lyophilized to dryness. The authenticity of the products was verified by MS (ESI-TOF) (Figures S1 and S2). Isolated yields (45–50%) of **C1** and **C2** were determined by UV absorbance at 260 nm.

#### Assembly of Amino-Modified [NH_2_]MSNA(antiHER2), [NH_2_]MSNA(scramble), and [NH_2_]MSNA(antiHER2-Cy5)

MSNAs were assembled as follows: **C1** or **C2** (100 nmol in 200 μL of H_2_O) was mixed with BCN–ON, **ON3**, **ON4**, or **ON5** (1200 nmol in 400 μL of H_2_O), and 257 μL of 5 M NaCl was added. The reaction mixture was gently shaken for 3 days at room temperature and subjected to RP-HPLC. An analytical RP-HPLC column Phenomenex, Aeris 3.6 μm WIDEPORE XB-C18 200 Å, 150 × 4.6 mm, linear gradient from 5% to 35% MeCN in 50 mmol L^–1^ of triethylammonium acetate over 25 min, a flow rate of 1.0 mL min^–1^, and detection at 260 nm were used for purification. The product fractions were collected and lyophilized to dryness. Isolated yields (40–50%) of the products were determined by UV absorbance at 260 nm. The homogeneity of **[NH**_**2**_**]MSNA(antiHER2)**, **[NH**_**2**_**]MSNA(scramble)**, and **[NH**_**2**_**]MSNA(antiHER2-Cy5)** was evaluated by PAGE (Figure S3).

### Synthesis of TCO-PEG_4_-Funtionalized MSNAs [TCO]MSNA(antiHER2), [TCO]MSNA(scramble), and [TCO]MSNA(anti-HER2-Cy5)

Succinimidyl ester of *trans*-cyclooctene-PEG_4_ ((2,5-dioxopyrrolidin-1-yl) 3-[2-[2-[2-[2-[[(4*Z*)-cyclooct-4-en-1-yl]oxycarbonylamino]ethoxy]ethoxy]ethoxy]ethoxy]propanoate, 1 μmol in 10 μL of DMSO) was added to a mixture of **[NH**_**2**_**]MSNA(antiHER2/scramble/antiHER2-Cy5)** (20 nmol each) in aqueous 0.1 M sodium borate (100 μL, pH 8.5), and the mixture was gently shaken for 4 h at rt. Phosphate-buffered saline PBS (450 μL) was added, and the excess TCO-PEG_4_-NHS was removed by centrifugal filtration (Amicon Ultra 30K MWCO, 9 min at 14000 g). The PBS addition and centrifugation were repeated five times. **[TCO]MSNA(antiHER2)**, **[TCO]MSNA(scramble)**, and **[TCO]MSNA(antiHER2-Cy5)** were recovered in 95% yield after the final centrifugation (based on UV absorbance at λ = 260 nm). The authenticity and homogeneity of **[TCO]MSNA(antiHER2)**, **[TCO]MSNA(scramble)**, and **[TCO]MSNA(antiHER2-Cy5)** were verified by PAGE (Figure S3) and SEC-MALS (Figures S4–S6).

### Tetrazine-Modified Antibodies

General procedure: Ab (**Tra** or **IgG**) glycan chains were first azide functionalized using a Genovis GlyClick Azide Activation kit following the manufacturer’s protocol. The azide-activated Ab (2 nmol in 50 μL of PBS) and Tz-DBCO (3-[[4-(2-azatricyclo[10.4.0.04,9]hexadeca-1(16),4,6,8,12,14-hexaen-10-yn-2-yl)-4-oxobutanoyl]amino]-1-[3-[4-(6-methyl-1,2,4,5-tetrazin-3-yl)phenoxy]propylamino]-1-oxopropane-2-sulfonic acid, 200 nmol in 2 μL of DMSO) were mixed and incubated for 4 h at rt. PBS (450 μL) was added, and the excess Tz-DBCO was removed by a centrifugal filtration (Amicon Ultra 30K MWCO, 9 min at 14000*g*). The PBS addition and centrifugation were repeated five times. **Tra-Tz** and **IgG-Tz** were recovered in ca. 90% yield after final centrifugation (based on UV-absorbance at 280 nm).

### Preparation of Ab–MSNA Conjugates

**Figure 1:**
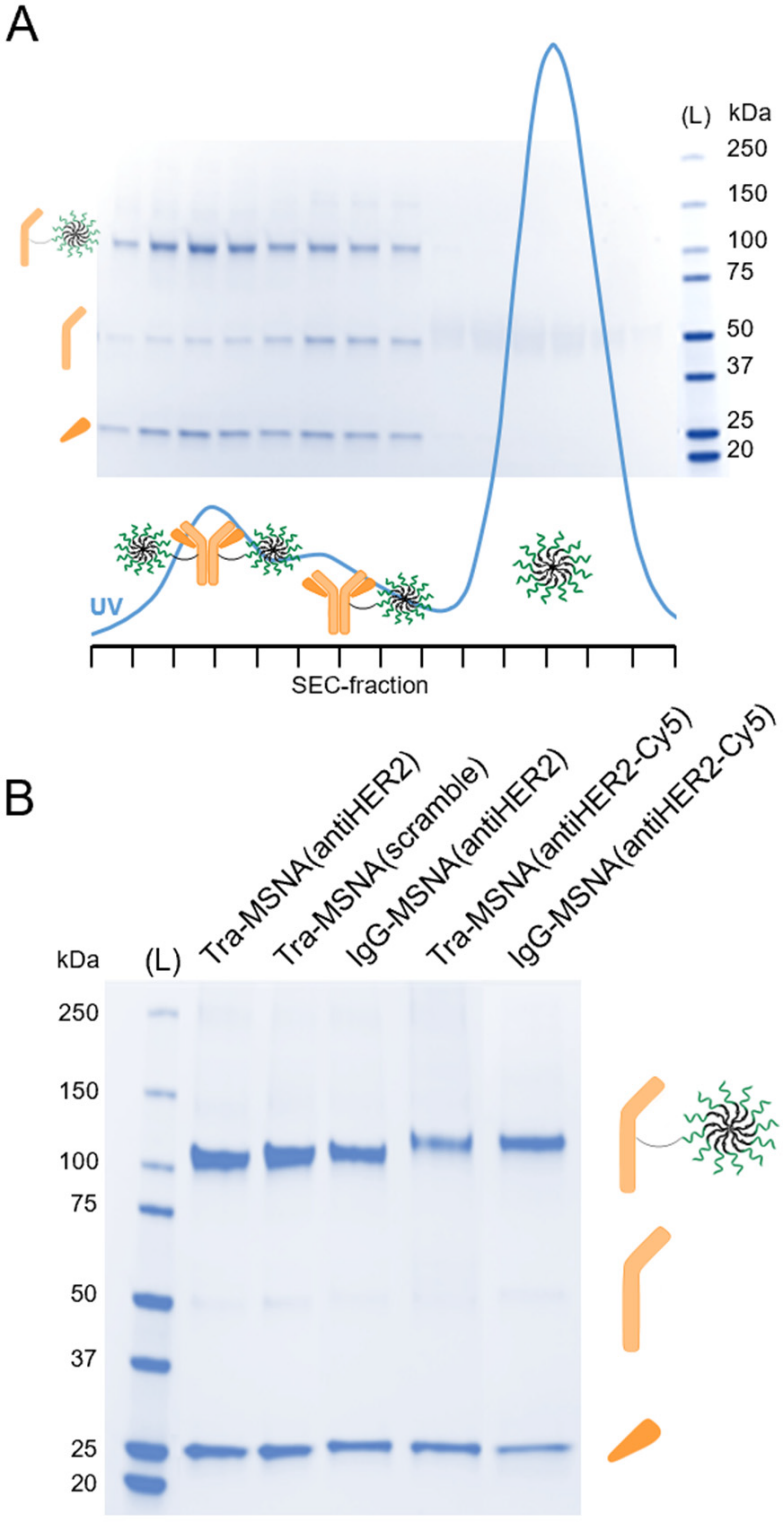
(A) Purification of Ab–MSNA conjugates [**Tra-Tz** + **[TCO]MSNA(antiHER2)** as an example]. UV profile from SEC purification of the crude product (blue line) and SDS-PAGE analysis of the corresponding fractions overlapped. (B) SDS-PAGE analysis of the synthesized Ab–MSNA conjugates. Molecular weight ladder. Corresponding denaturation products are annotated next to gels.

**Figure 2:**
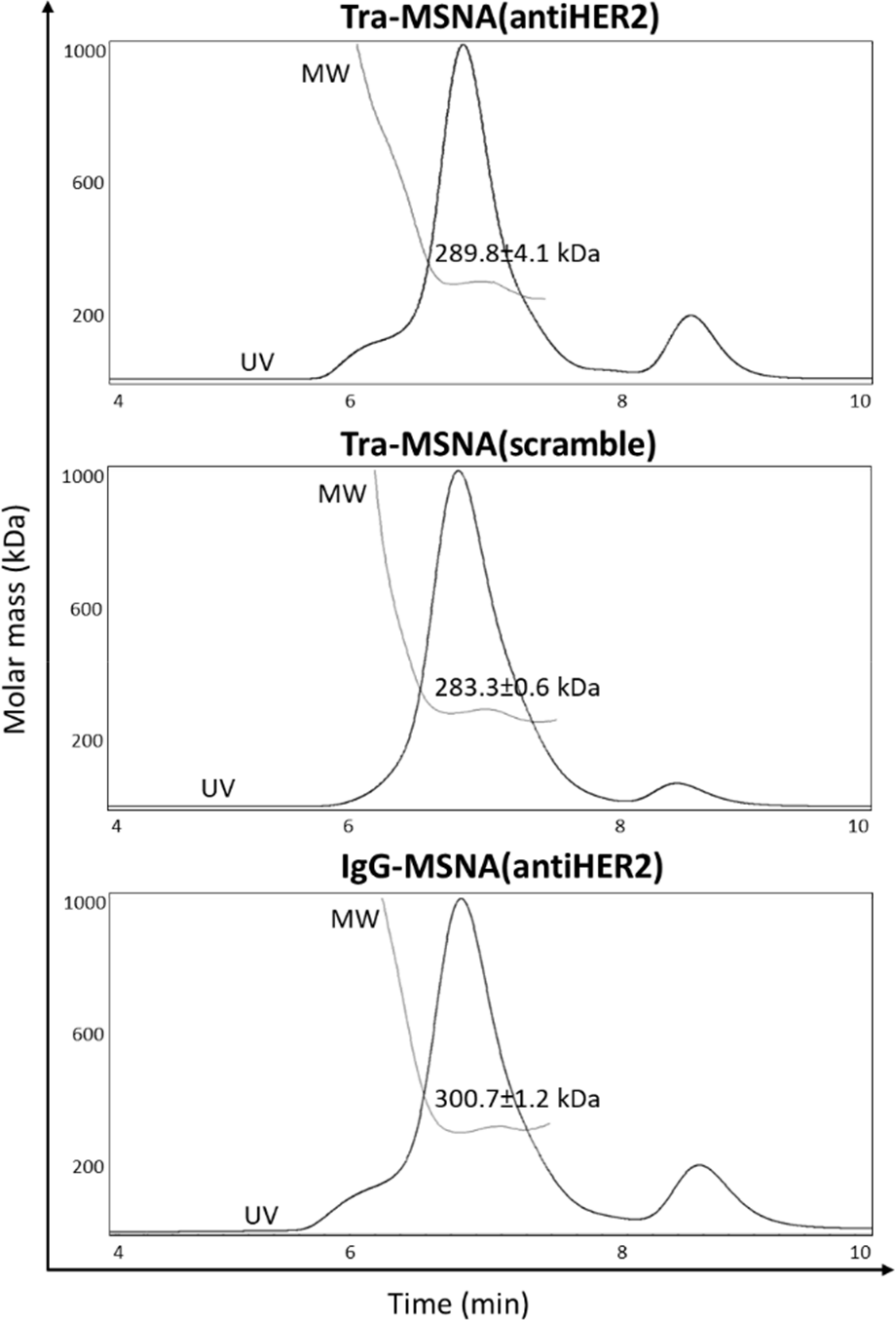
SEC-MALS analysis of the Ab–MSNA conjugates. Expected molecular weights for **Tra-MSNA(antiHER2)** and **Tra-MSNA(scramble)**: 270.7 kDa (both) and for **IgG-MSNA**: 268.7 kDa.

General procedure: Tetrazine-modified antibody (1 nmol in 10 μL of PBS) and TCO-modified MSNA (8 nmol in 80 μL of PBS) were mixed and incubated at room temperature for 4 h. The mixture was purified with size exclusion chromatography using a Superdex 200 Increase 10/300 GL column (isocratic elution with phosphate-buffered saline pH 7.4 at a flow rate of 0.75 mL min^–1^). Fractions were analyzed with SDS-PAGE, and those containing the favored 1:2 ratio Ab–MSNA conjugate were pooled. **Tra-MSNA(antiHER2)**, **Tra-MSNA(scramble)**, **Tra-MSNA(antiHER2-Cy5)**, **IgG-MSNA(antiHER2)**, and **Tra-MSNA(antiHER2-Cy5)** were obtained in 20–26% yield (based on UV absorbance at λ = 260 nm) after SEC purification and analyzed with SDS-PAGE (Figure 1B) and SEC-MALS (Figure 2).

### Cyanine5-Labeled Trastuzumab

Succinimidyl ester of sulfo-cyanine 5 1-[6-(2,5-dioxopyrrolidin-1-yloxy)-6-oxohexyl]-3,3-dimethyl-2-[(1*E*,3*E*,5*E*)-5-(1,3,3-trimethyl-5-sulfonatoindolin-2-ylidene)penta-1,3-dienyl]-3*H*-indolium-5-sulfonate, in DMSO, 10 nmol, 20 equiv) was added to a mixture of **Tra** (0.5 nmol) in 0.1 M aqueous sodium borate (pH 8.5), and the mixture was incubated for 4 h at rt. **Tra-Cy5** was purified twice with a spin desalting column (7 K MWCO) and recovered in 85% yield based on absorbance at 280 nm. UV–vis absorbance at 280 and 646 nm was used to determine the ratio of Tra/Cyanine5 (degree of labeling = 5.8).

### PAGE Analysis of the MSNAs

Native 6% Tris base, boric acid, EDTA, and acrylamide (TBE) gel were used to analyze the MSNAs’ purity. A precast gel cover (10 cm × 10 cm in size, Thermo Fisher Scientific) was fixed into a vertical electrophoresis chamber, and the running buffer (90 mM Tris, 90 mM borate, and 2 mM EDTA, 8.3 pH) was filled into the chamber. MSNA samples (0.5 pmol in 5 μL of PBS) mixed with 5 μL of TBE sample buffer and a DNA ladder (100, 200–1000 bp; note, the ladder is just used to confirm the quality and comparability of the runs and cannot be used for size evaluation of the MSNAs) were loaded and electrophoresed at constant 200 V for approximately 30 min. After completion of electrophoresis, gel was removed from the chamber and stained by SYBR Gold Nucleic Acid Stain (Thermo Fisher Scientific) for 1 h prior to imaging with the Gel Doc imaging system (Bio-Rad).

### SEC-MALS Experiments

SEC-MALS was performed using an Agilent Technologies 1260 Infinity II HPLC system (sampler, pump, and UV–vis detector) equipped with a Wyatt Technologies miniDAWN light-scattering detector and Wyatt Technologies Optilab refractive index detector. An Agilent AdvanceBio SEC 300 Å, 2.7 μm, 4.6 × 300 mm column and 150 mM sodium phosphate, pH 7.0, as the mobile phase eluting at a rate of 0.2 mL min^–1^ and run time of 20 min were used for each experiment (injected sample concentrations and volumes: 1 mg mL^–1^ in 20 μL of PBS for MSNAs and 0.1 mg mL^–1^ in 50 μL of PBS for Ab-MSNAs). Molecular weights were determined using a refractive index increment (d*n*/d*c*) of 0.1703 mL/g.

### SDS-PAGE Analysis of Conjugates

Denaturating polyacrylamide gel electrophoresis was used to analyze the Ab–MSNA conjugates. A precast gel cover (4–15% Mini-PROTEAN TGX Precast Protein, Bio-Rad) was fixed into a vertical electrophoresis chamber, and the running buffer (25 mM Tris, 192 mM glycine, and 0.1% SDS, pH 8.3) was filled into the chamber. Samples (mixed with 4× Laemmli Sample buffer, 10% 2-mercaptoethanol) and a protein ladder (Precision Plus Protein Dual Color Standard, Bio-Rad) were loaded and electrophoresed at 200 V for approximately 30 min. After completion of electrophoresis, gel was removed from the chamber and stained with Readyblue Protein Gel Stain (Sigma-Aldrich) for 2 h prior to imaging with a Gel Doc imaging system (Bio-Rad).

### Cell Culture

BT-474 cells were purchased from the American Tissue Culture Collection (ATCC) and grown in Gibco DMEM, low glucose, GlutaMAX, pyruvate medium (Thermo Fisher Scientific) supplemented with 10% fetal bovine serum (FBS) and 0.07% insulin. The cells were detached from the T75 flask using 0.25% trypsin. After the cells were aliquoted to new flasks in a 1:3 ratio, the cell culture was maintained at 37 °C with 5% CO_2_.

### Cellular Experiments

BT-474 cells were seeded at 30% confluency on a 96-well plate 24 h before experiments. For internalization assay, Cy5-labeled test items were diluted with Gibco RPMI 1640 (without phenol red) at 20 nM concentration. Cells were subjected to the prepared solutions, and after 30 min incubation on ice the cells were washed twice with ice cold PBS. A fresh medium was applied, and intracellular delivery of compounds was monitored for 16 h at 2 h intervals via fluorescent microscopy on 649 nm using 37 °C and a 5% CO_2_ live-cell imaging instrument (Operetta, PerkinElmer). Cells were visualized with digital phase contrast.

For proliferation assay, cells were subjected to test items at 0.62, 1.85, 5.56, 16.67, and 50 nM final concentrations and monitored for 72 h at 4 h intervals via phase contrast microscopy on a 37 °C, 5% CO_2_ live-cell imaging instrument (IncuCyte, Sartorius). Confluency values (Figure 5) were calculated from images (Figure S7) by using software provided with the instrument.

### Biolayer Interferometry Experiments

Experiments were conducted with FortéBio’s Octet RED96e biolayer interferometry (BLI) biosensor instrument. Assay method one: The biotinylated HER2 was loaded into streptavidin (SA) sensors (Sartorius) for 300 s. HER2-loaded SA sensors were dipped into 1× Octet kinetics buffer (Sartorius) for 60 s to establish a baseline, followed by exposure to each analyte solution (11.1–100 nM in Octet kinetics buffer) for 300 s for association and then dissociation in kinetics buffer for 600 s. Assay method two: **Tra**, **Tra-MSNA(antiHER2)**, and **Tra-MSNA(scramble)** were loaded to anti-humanIgG FC capture (AHC) sensors (Sartorius) for 300 s. Loaded AHC sensors were dipped into 1× Octet kinetics buffer for 60 s to establish a baseline, followed by exposure to HER2 solution (0.93–75 nM in kinetics buffer) for 300 s for association and then dissociation in kinetics buffer for 600 s.

## Results and Discussion

### Synthesis of TCO-Modified MSNAs

BCN-modified ONs used for the assembly of MSNAs were synthesized with an automated synthesizer using commercial phosphoramidite building blocks and conventional coupling chemistry. **ON1**, **ON3**, and **ON4** contain an antisense sequence, the activity of which in SNA formulation has previously been demonstrated to down regulate HER2.^27,30^ SPAAC between an azide-modified [60]fullerene **1** and BCN-modified ONs was used for the assembly of amino-modified MSNAs (**[NH**_**2**_**]MSNA(antiHER2)**, **[NH**_**2**_**]MSNA(scramble)**, and **[NH**_**2**_**]MSNA(antiHER2-Cy5)**, Scheme 1) following a two-step synthesis protocol:^33^ First, 3′-amino- and 5′-BCN-modified ONs (**ON1**, **ON2**) were treated with an excess of **1** (4 equiv) in DMSO to give [60]fullerene–ON conjugates **C1** and **C2** in 45% and 50% isolated yield. Then, **C1** and **C2** were exposed to a slight excess (1.2 equiv/azide arm) of 5′-BCN-modified ONs (**ON3**–**ON5**) in 1.5 mol L^–1^ aqueous NaCl to yield **[NH**_**2**_**]MSNA(antiHER2)**, **[NH**_**2**_**]MSNA(scramble)**, and **[NH**_**2**_**]MSNA(antiHER2-Cy5)** in 40–50% isolated yield. The homogeneity of [NH_2_]MSNAs was confirmed by PAGE (Figure S3), and it was introduced to amide coupling with an *N*-hydroxysuccinimidyl (NHS) ester-activated TCO reagent (for 4 h at rt). Spin column filtration (30 kDa molecular weight cutoff) afforded the products: **[TCO]MSNA(antiHER2)**, **[TCO]MSNA(scramble)**, and **[TCO]MSNA(antiHER2-Cy5)** in 95% recovery. The homogeneity and authenticity of the products were confirmed by PAGE (Figure S3) and SEC-MALS (Figures S4–S6). The SEC-MALS-based molecular weight analysis of the products matched well with the expected molecular masses: **[TCO]MSNA(antiHER2)**, **[TCO]MSNA(scramble)**, and **[TCO]MSNA(antiHER2-Cy5)**: 59.6 ± 0.9 kDa (expected 61.5 kDa), 59.2 ± 0.9 kDa (expected 61.5 kDa), and 78.1 ± 0.6 kDa (expected 70.2 kDa), respectively.

### Synthesis of Tetrazine-Modified Ab

Glycan chains of **Tra** and **IgG** were azide-modified with an established and commercially available kit (GlyCLICK, Genovis) that covers two successive enzymatic reactions: First, the glycan chain is deglycosidated from the innermost *N*-acetylglucosamine (GlcNAc) residue with a specific endoglycosidase. Second, uridine diphosphate *N*-azidoacetylgalactosamine (UDP-GalNAz) as a substrate is attached to the exposed GlcNAc residue using galactosyltransferase, yielding the site-specifically azide-modified Ab (two azides/Ab). Azide-modified Abs (**Tra-N**_**3**_ and **IgG-N**_**3**_, Scheme 1) were then treated with an excess (100 equiv) of a bifunctional methyltetrazine/dibenzocyclooctyne linker (**Tz-DBCO**) (for 4 h at rt) and purified by spin column filtration (50 kDa molecular weight cutoff) to yield tetrazine-modified Abs: **Tra-Tz** and **IgG-Tz**.

### Synthesis of Ab–MSNA Conjugates

The tetrazine-modified Abs **Tra-Tz** and **IgG-Tz** have two reactive tetrazine groups, one in both heavy chains. An excess (8 equiv) of the [TCO]MSNAs was used to drive the iEDDA conjugation to 1:2 Ab–MSNA species. The [TCO]MSNAs and the tetrazine-modified Abs were incubated in PBS for 4 h at rt; the reaction mixtures were subjected to SEC; and the eluted fractions were analyzed by SDS-PAGE (Figure 1A: **Tra-Tz** + **[TCO]MSNA(antiHER2)** as an example). Each reaction mixture contained the 1:2-Ab–MSNA conjugate as a major product and the 1:1 Ab–MSNA conjugate as a minor product. The 1:2 Ab–MSNA conjugates were isolated by SEC in 20–26% yields. As seen in SDS-PAGE (Figure 1B), homogeneous products were obtained despite the partial overlapping of the product fractions in SEC (Figure 1A). SDS-PAGE could be used to evaluate the authenticity of the products. Migration of the reduced products represented the expected species of MSNA-conjugated heavy chains at 110 and 120 kDa for nonlabeled and cyanine-5-labeled conjugate, respectively (Figure 1B). SEC-MALS was applied to assess further the homogeneity and molecular weights of the nonlabeled Ab–MSNA conjugates, prepared in a larger quantity. The SEC-MALS-based molecular weight analysis of these hybrid conjugates showed somewhat modest accuracy (Figure 2), but it, together with the SDS-PAGE analysis, could be used to assess the authenticity of the products. In overall, five different Ab–MSNA conjugates were prepared: **Tra-MSNA(antiHER2)**, **Tra-MSNA(scramble)**, **Tra-MSNA(antiHER2-Cy5)**, **IgG-MSNA(antiHER2)**, and **IgG-MSNA(antiHER2-Cy5)**.

### Antigen-Binding Experiments with Biolayer Interferometry

**Figure 3:**
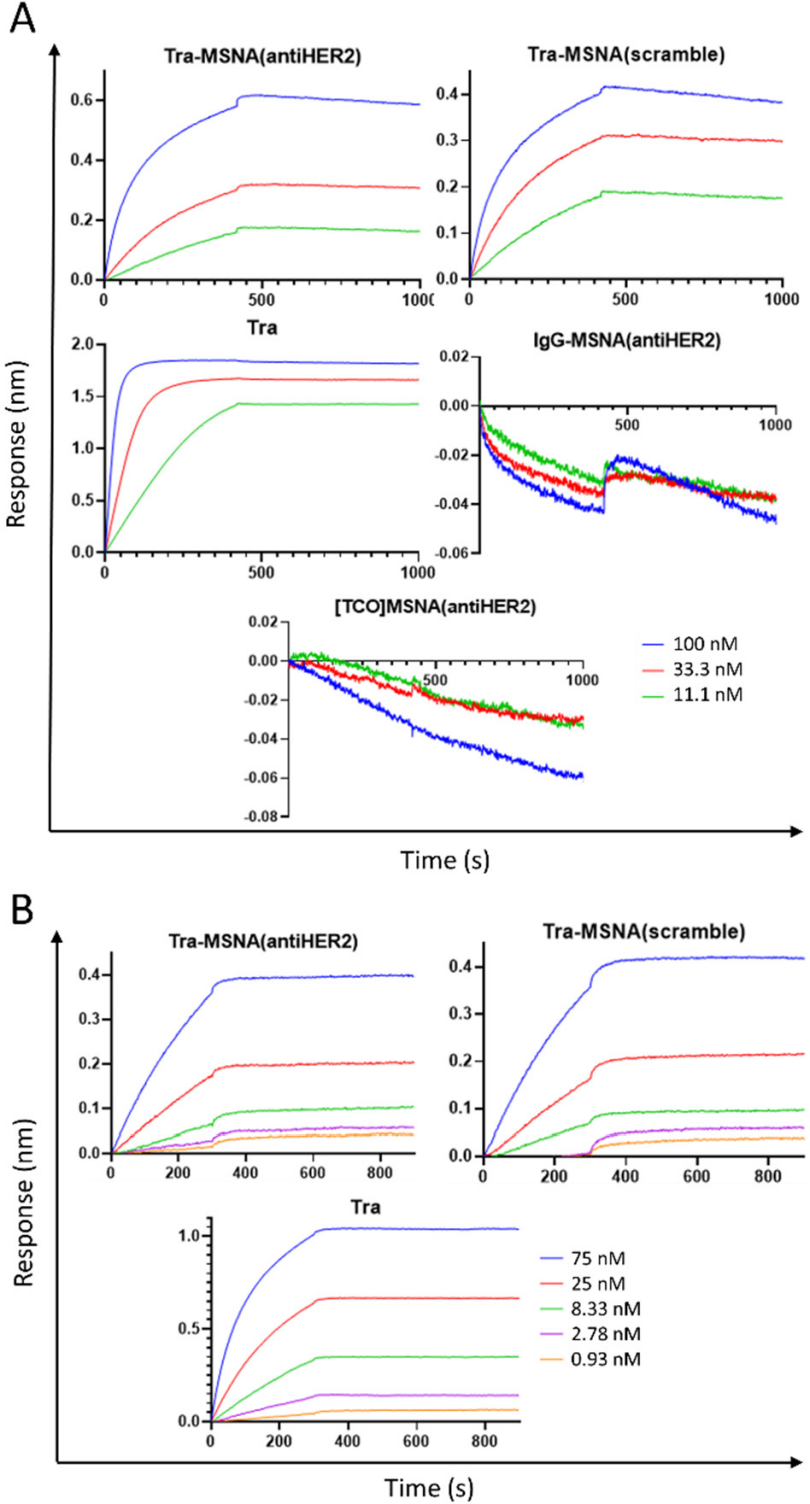
Biolayer interferometry (BLI) experiment to study HER2 binding of Ab–MSNA conjugates. (A) Biotinylated HER2 immobilized on a streptavidin sensor and exposed to **Tra**, **Tra-MSNA(antiHER2)**, **Tra-MSNA(scramble)**, **IgG-MSNA(antiHER2)**, and **[TCO]MSNA(antiHER2)** as analytes. (B) **Tra**, **Tra-MSNA(antiHER2)**, and **Tra-MSNA(scramble)** immobilized on anti-hIgG Fc capture sensor and exposed to HER2 as an analyte. Association 300 s and dissociation 600 s for both experiments.

In general, site-specific conjugation to an Ab’s Fc region does not alter the immuno-reactivity.^41,42^ However, we expected that the sterically demanding negatively charged MSNA payload might have an effect on the binding properties of the Ab constituent. To evaluate how the MSNA constituent affects Tra’s affinity toward the HER2 protein, biolayer interferometry (BLI) experiments were carried out. Biotinylated HER2 protein was immobilized in a streptavidin sensor. **Tra**, **Tra-MSNA(antiHER2)**, **Tra-MSNA(scramble)**, **IgG-MSNA(antiHER2)**, and **[TCO]MSNA(antiHER2)** were used as analytes in 100, 33, and 11 nM concentrations (Figure 3A). **Tra** and its MSNA conjugates were found to bind to the sensor in a concentration-dependent manner. **IgG-MSNA(antiHER2)** and **[TCO]MSNA(antiHER2)** did not bind to the sensor, indicating that the binding was Tra-mediated. To further investigate this matter, we reversed the assay and immobilized **Tra**, **Tra-MSNA(antiHER2)**, and **Tra-MSNA(scramble)** to anti-hIgG Fc capture sensors and used free HER2 protein as an analyte in 75 25, 8.3, 2.8, and 0.93 nM concentrations (Figure 3B). This assay supported the findings of the initial experiment; **Tra** and its MSNA conjugates all had affinity toward HER2 protein, and the binding was concentration-dependent in each case. However, the binding response of **Tra-MSNA(HER2)** and **Tra-MSNA(scramble)** was approximately one-third of that of **Tra**. Despite the reduced response, which is likely to occur with high ON-payload–Ab conjugates, BLI experiments demonstrated that the Tra–MSNA conjugates retained the Tra-mediated activity toward HER2.

### Internalization Studies

**Figure 4:**
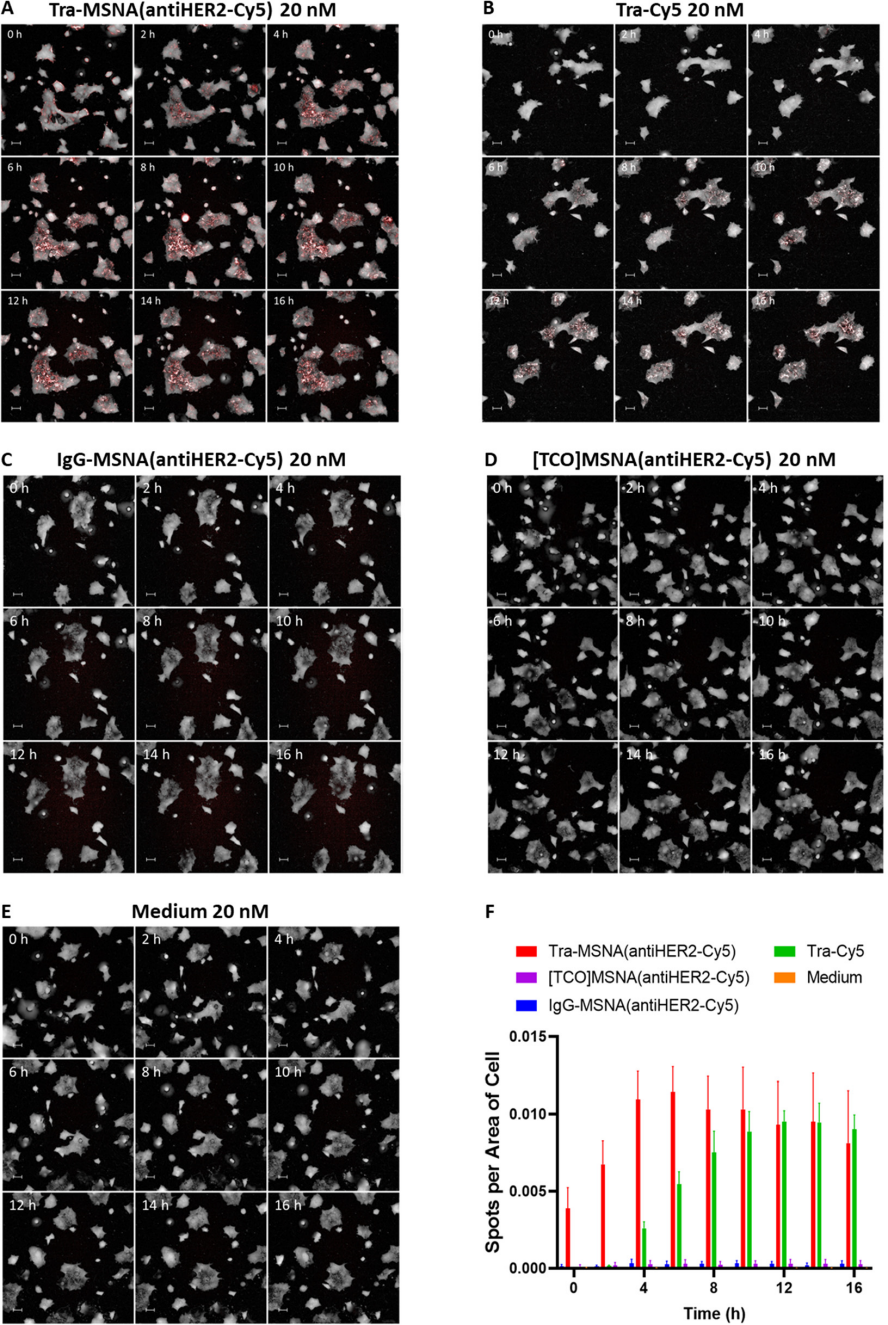
Internalization of BT-474 cells. Prior to imaging, cells were incubated for 30 min on ice with 20 nM solution of cyanine 5-labeled compounds, after which unbound compounds were washed away with ice cold PBS for time points of 0–16 h and incubation at 37 °C. (A–E) Channels 647 nm (red) and digital phase contrast combined for representable time series images for internalization. The scale bar is 50 μm. (F) Average number of fluorescent spots (±SD) detected from the 647 nm (red) channel per cell detected on phase contrast. Data combined from two replicates.

Binding properties could be evaluated by the complementary BLI experiments above. However, these experiments, based on either immobilized Tra or immobilized HER2, do not guarantee the functionality of the HER2-Tra-mediated endocytosis. HER2-receptor-mediated cellular uptake of the cyanine 5-labeled conjugates on BT-474 breast carcinoma cells was monitored by fluorescent microscopy at λ = 649 nm (Figure 4). Cells were incubated on ice in 20 nM solutions of **Tra-MSNA(antiHER2-Cy5)**, **IgG-MSNA(antiHER2-Cy5)**, **[TCO]MSNA(antiHER2-Cy5)**, and **Tra-Cy5**. After 30 min incubation, unbound compounds were washed off with ice cold PBS. Cells were then imaged for 16 h in 2 h intervals in a 37 °C, 5% CO_2_ live-cell imaging instrument. Digital phase contrast was used to visualize cells. At low temperatures, only the species with affinity toward HER2 are bound to the cell surface.^43^ Increased temperature (37 °C) initiates endocytosis of the HER2–Tra-MSNA complex. With **Tra-MSNA(antiHER2-Cy5)** and **Tra-Cy5**, the fluorescence signal is seen on the edges of the cells at early time points. As the study advanced, the fluorescence signal moved toward the middle of the cell, indicating that compounds were internalized. No fluorescence signal was observed with **IgG-MSNA(antiHER2-Cy5)** and **[TCO]MSNA(antiHER2-Cy5)**. Quantitation of fluorescent spots per cell (Figure 4F) supported the visual observations from the microscope images. As time progressed, the fluorescent signal moved from the edges and spread toward the inside of the cell, leading to more countable spots in cells treated with Tra and its MSNA conjugate. Interestingly, **Tra-Cy5** and **Tra-MSNA(antiHER2-Cy5)** produced different kinetic profiles as the MSNA conjugate seemed to be internalized more rapidly (Figure 4F). However, no real comparison of their internalization kinetics can be made from these results since the compounds are not analogous regarding labeling site and degree of labeling. The findings from the internalization assay suggest that HER2-mediated endocytosis works with the high ON payload–Ab conjugates and supports the results of BLI experiments regarding the retained binding affinity.

### Proliferation Assay

**Figure 5:**
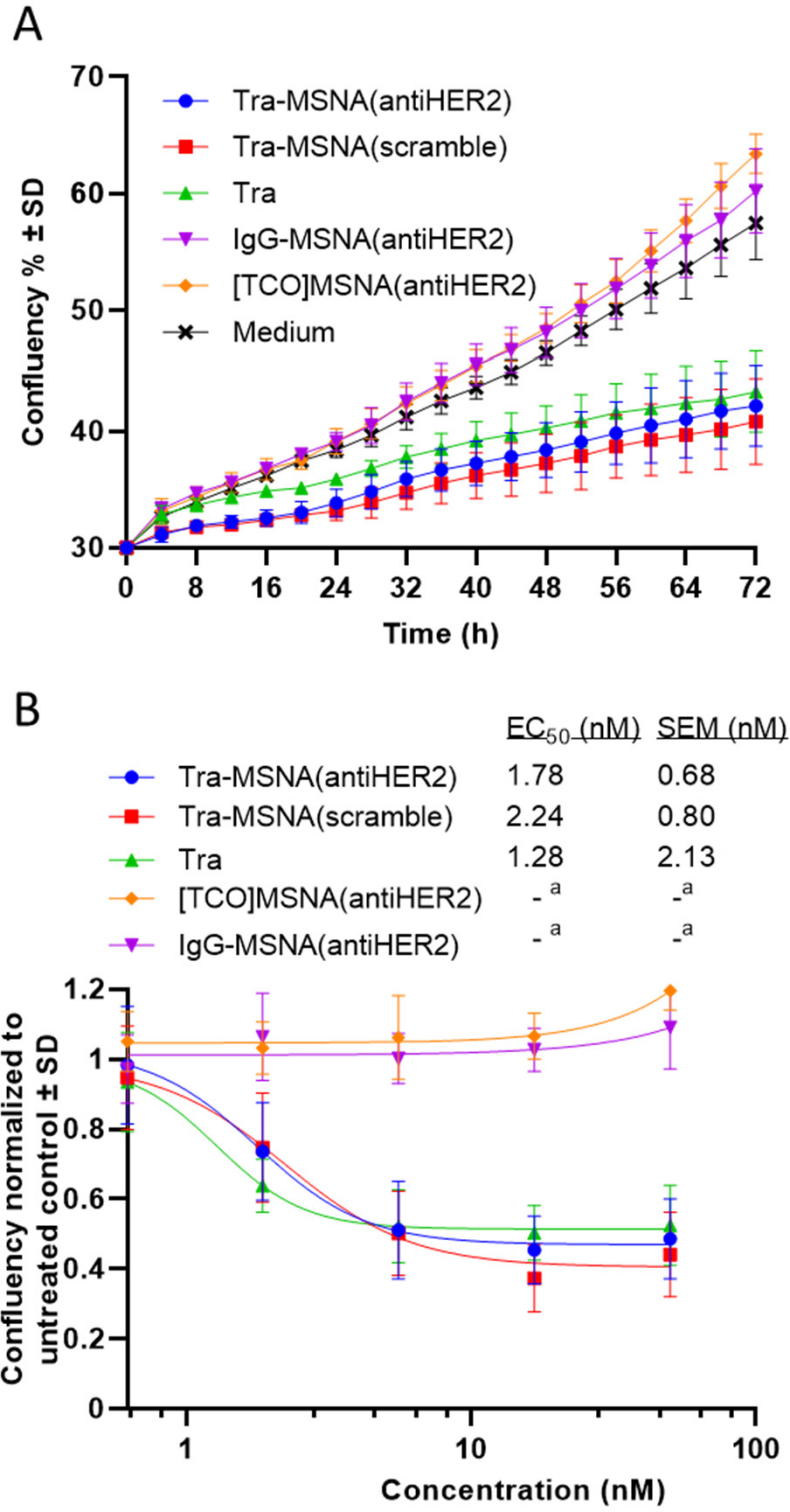
Proliferation of BT-474 cells. (A) Cells were incubated with 50 nM final concentration of compounds and imaged using phase contrast for every 4 h for 72 h. Confluency was plotted against time. (B) Dose–response from a 72 h time point normalized to untreated control (= 1). Half maximal effective inhibitory concentration (EC_50_) was calculated from the fitted dose–response curves. All measured points are the average ± SD of four replicates. ^*a*^EC_50_ value could not be determined.

To investigate the effect of the conjugates on the proliferation of the BT-474 breast cancer cells, label-free live-cell time-lapse imaging was utilized. Cells were treated with 0.62–50 nM solutions of **Tra-MSNA(antiHER2)**, **Tra-MSNA(scramble)**, **Tra**, **IgG-MSNA(antiHER2)**, and **[TCO]MSNA(antiHER2)** and imaged for 72 h in 4 hour intervals in a 37 °C, 5% CO_2_ live-cell imaging instrument. As seen in Figure 5, conjugates containing Tra inhibited the proliferation of the HER2-positive BT-474 cells, whereas **[TCO]MSNA(antiHER2)** and **IgG-MSNA(antiHER2)** had no effect on cell proliferation. A marginal difference between profiles of **Tra**, **Tra-MSNA(antiHER2)**, and **Tra-MSNA(scramble)** demonstrates that the ON constituent had virtually no effect on the proliferation, which indicates that all activity comes from the binding of Tra to HER2. Half maximal effective concentration (EC_50_) of the compounds was calculated from the 72 h time point; EC_50_ for **Tra-MSNA(antiHER2)** was 1.78 nM (SEM = 0.68 nM), for **Tra-MSNA(scramble)** 2.24 nM (SEM = 0.80 nM), and for **Tra** 1.28 nM (SEM = 2.13 nM), respectively. The similarity of dose–response profiles could be attributed to readily strong antiproliferative effects of **Tra** and furthermore to the cell’s limited capacity to regenerate HER2 receptors on its surface following Ab binding and endocytosis.^44^ In addition, the stable covalent linkage of the model conjugate **Tra-MSNA(antiHER2)** might interfere with delivery of the antiHER2-ON payload to cytoplasm and, consequently, downregulation of HER2.^45^ Despite the lack of improvement in the antiproliferation properties, the proliferation assay supported the findings of the BLI and internalization experiments: The model high ON–payload Ab conjugates **Tra-MSNA(antiHER2)** managed to retain the Tra-HER2 response.

## Conclusions

The scope of this article was to describe molecularly uniform conjugates of molecular spherical nucleic acids and antibodies for the first time. The conjugates were synthesized in a molecularly defined and site-specific manner via biocompatible iEDDA-based conjugation between [60]fullerene-based molecular spherical nucleic acids and a glycan-engineered antibody. The desired conjugates (antibody–oligonucleotide ratio of 1:24) were isolated in relatively high yield and purity. The conjugates were found to bind to the target antigen in a concentration-dependent manner, as studied by biolayer interferometry. Receptor-mediated internalization on BT-474 breast carcinoma cells was demonstrated with fluorescently labeled conjugates, and the effect of the Trastuzumab-containing compounds on BT-474 cell proliferation was shown. This promising data on retained antibody-mediated targeting potential and the antiproliferation effect of these high oligonucleotide payload–antibody conjugates would suggest further studies with a cleavable linker design^46^ or, alternatively, evaluation of more active oligonucleotide payloads for more potent antiproliferative effect with, e.g., G3139, which is an antisense oligonucleotide drug that targets Bcl-2 mRNA and induces cell apoptosis.^47^ This framework could also be utilized for conjugating antibodies to dendritic oligonucleotides without the full architecture of spherical nucleic acids.^48^ Overall, this work introduces novel uniform and site-specific high payload oligonucleotide antibody conjugates that could find diagnostic and therapeutic applications.

## Supporting Information Available

Characterization data (ESI-TOF, SEC-MALS, PAGE) of MSNAs and their intermediates and selected microscopy images from proliferation assays (PDF)
