# Supplementary material for: Synthesis of Site-Specific Antibody–[60]Fullerene–Oligonucleotide Conjugates for Cellular Targeting
Source: ACS Appl Bio Mater. 2023 Jul 11;6(8):3189–98. doi: 10.1021/acsabm.3c00318 (PMC10445261; doi:10.1021/acsabm.3c00318)
Supplement: Supplementary file 1 — mt3c00318_si_001.pdf [file mt3c00318_si_001.pdf]

Supporting information to:

Synthesis of site-specific antibody-[60]fullerene-oligonucleotide conjugates for cellular targeting

Antti Äärelä[a,b], Kati Räsänen[b], Patrik Holm[b], Harri Salo[b] and Pasi Virta[a*]

[a]Department of Chemistry, University of Turku, FI-20500 Turku, Finland

[b]Research and Development, Orion Pharma, FI-20380 Turku, Finland.

Email: pamavi@utu.fi

List of figures

Table S1 .... **S2**

Figure S1 .... **S3**

Figure S2 .... **S3**

Figure S3 .... **S4**

Figure S4 .... **S5**

Figure S5 .... **S5**

Figure S6 .... **S5**

Figure S7 .... **S6**

Structure		MS calc.	MS found
**ON1**	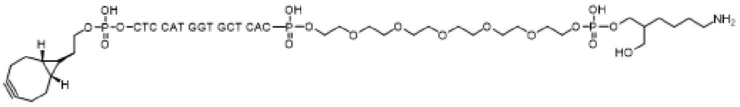	4971.09	4970.70
**ON2**	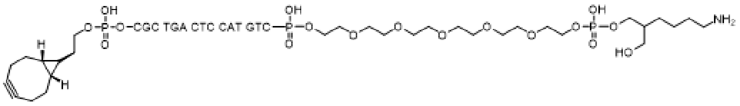	4971.09	4970.75
**ON3**	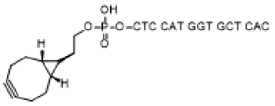	4730.18	4730.86
**ON4**	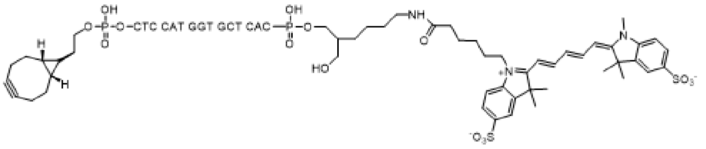	5563.12	5563.72
**ON5**	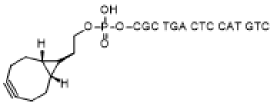	4730.18	4730.83

Table S1. Structures and MS (ESI-TOF) characterization of BCN-modified oligonucleotides used for MSNA assembly.

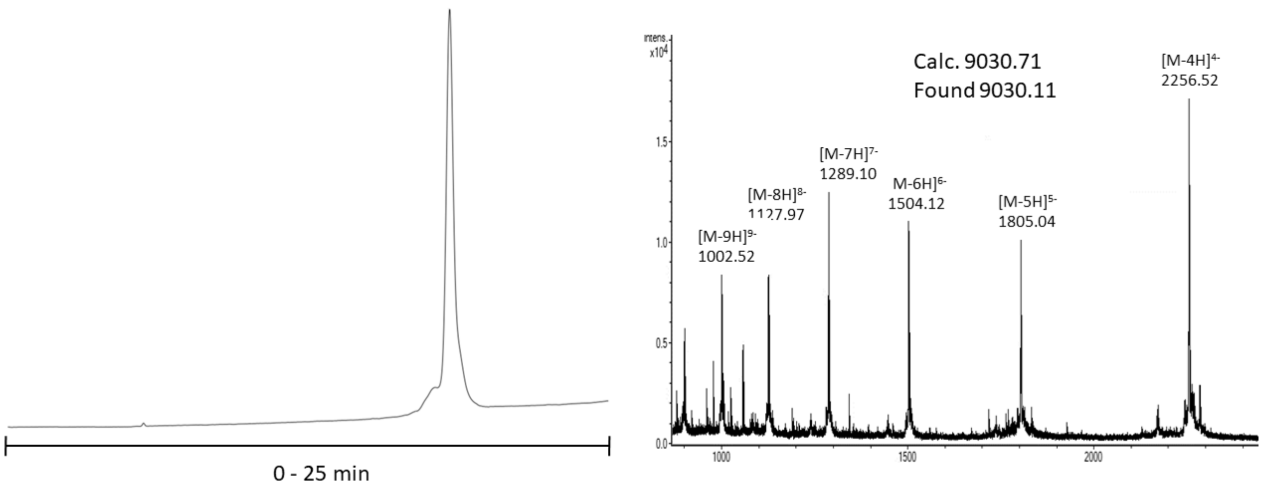

Figure S1. Structure and characterization of C60-ON conjugate **C1** A) RP HPLC profile and B) MS (ESI-TOF) spectrum. RP HPLC conditions: An analytical RP column (250 × 4.6 mm, 5 µm), detection at λ = 260 nm, gradient elution (0 - 25 min) from 40 to 100% MeCN in 50 mM triethylammonium acetate, flow rate 1.0 mL min^{-1}.

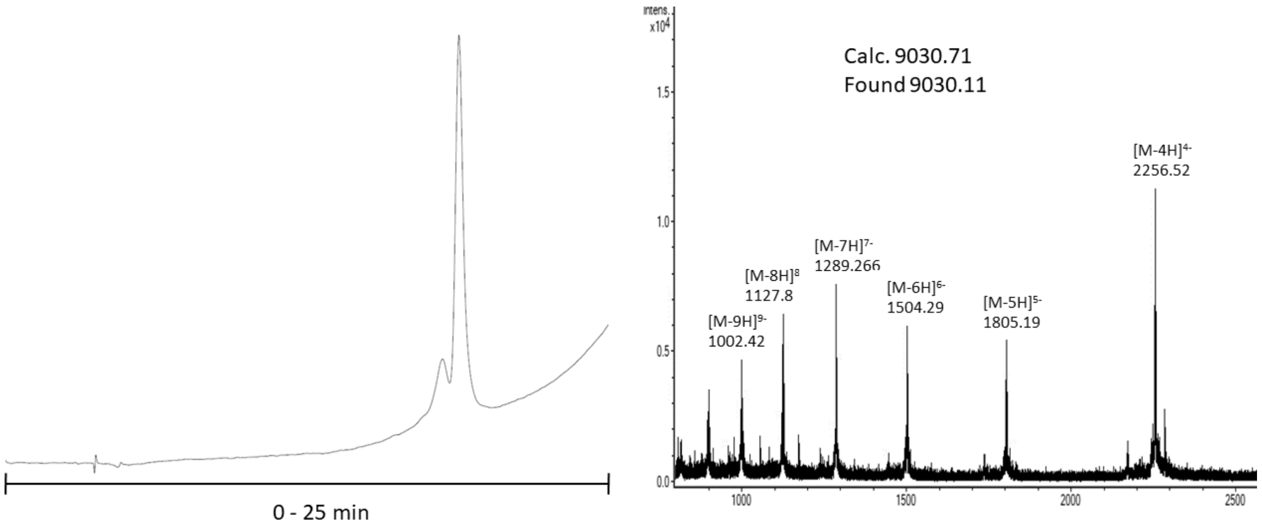

Figure S2. Structure and characterization of C60-ON conjugate **C2** A) RP HPLC profile and B) MS (ESI-TOF) spectrum. RP HPLC conditions: An analytical RP column (250 × 4.6 mm, 5 µm), detection at λ = 260 nm, gradient elution (0 - 25 min) from 40 to 100% MeCN in 50 mM triethylammonium acetate, flow rate 1.0 mL min^{-1}.

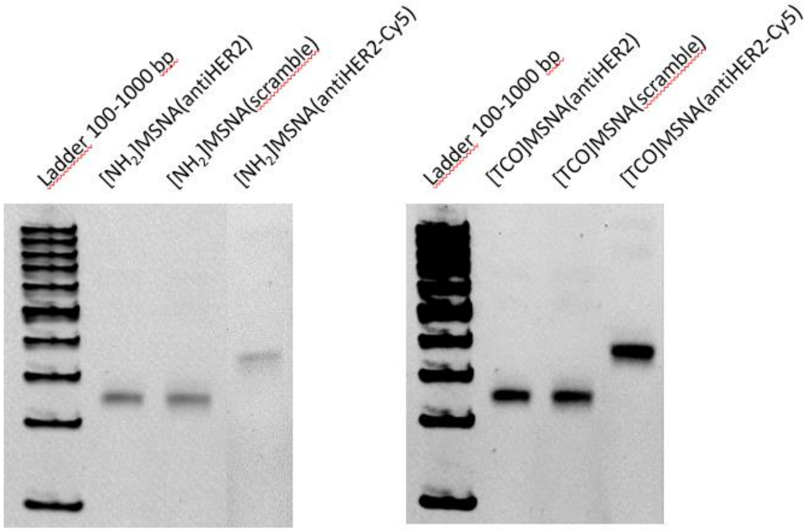

Figure S3. PAGE analysis of MSNAs.

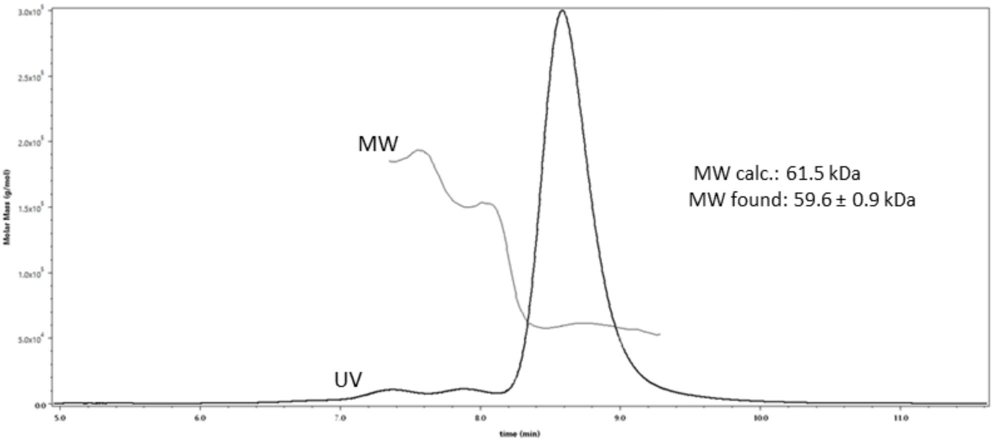

Figure S4. SEC-MALS profile of **[TCO]MSNA(antiHER2)**

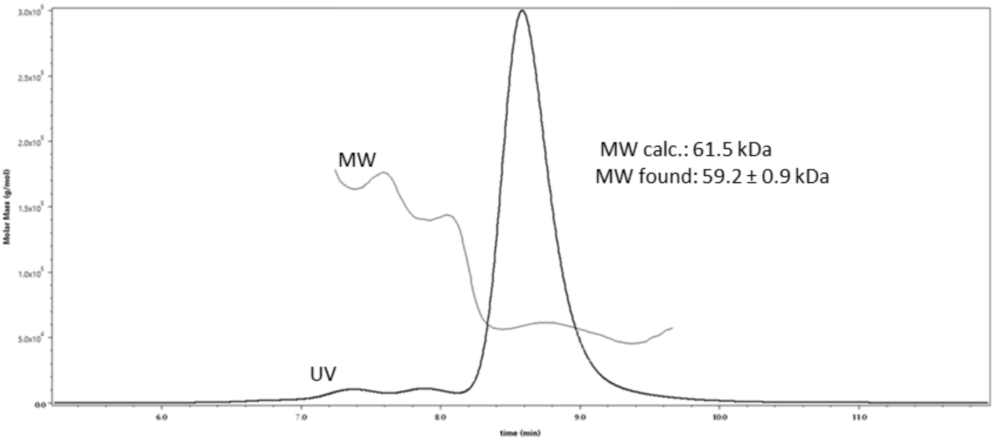

Figure S5. SEC-MALS profile of **[TCO]MSNA(scramble)**

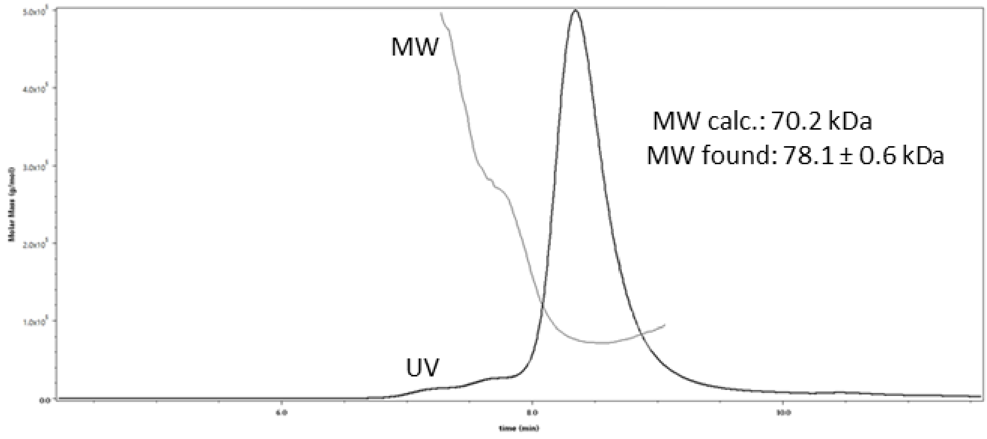

Figure S6. SEC-MALS profile of **[TCO]MSNA(antiHER2-Cy5)**

Tra-MSNA(antiHER2) 50 nM	Tra-MSNA(scramble) 50 nM	IgG-MSNA(antiHER2) 50 nM	Tra 50 nM	[TCO]MSNA(antiHER2) 50 nM
Tra-MSNA(antiHER2) 16.67 nM	Tra-MSNA(scramble) 16.67 nM	IgG-MSNA(antiHER2) 16.67 nM	Tra 16.67 nM	[TCO]MSNA(antiHER2) 16.67 nM
Tra-MSNA(antiHER2) 5.56 nM	Tra-MSNA(scramble) 5.56 nM	IgG-MSNA(antiHER2) 5.56 nM	Tra 5.56 nM	[TCO]MSNA(antiHER2) 5.56 nM
Tra-MSNA(antiHER2) 1.85 nM	Tra-MSNA(scramble) 1.85 nM	IgG-MSNA(antiHER2) 1.85 nM	Tra 1.85 nM	[TCO]MSNA(antiHER2) 1.85 nM
Tra-MSNA(antiHER2) 0.62 nM	Tra-MSNA(scramble) 0.62 nM	IgG-MSNA(antiHER2) 0.62 nM	Tra 0.62 nM	[TCO]MSNA(antiHER2) 0.62 nM
			Medium	Medium

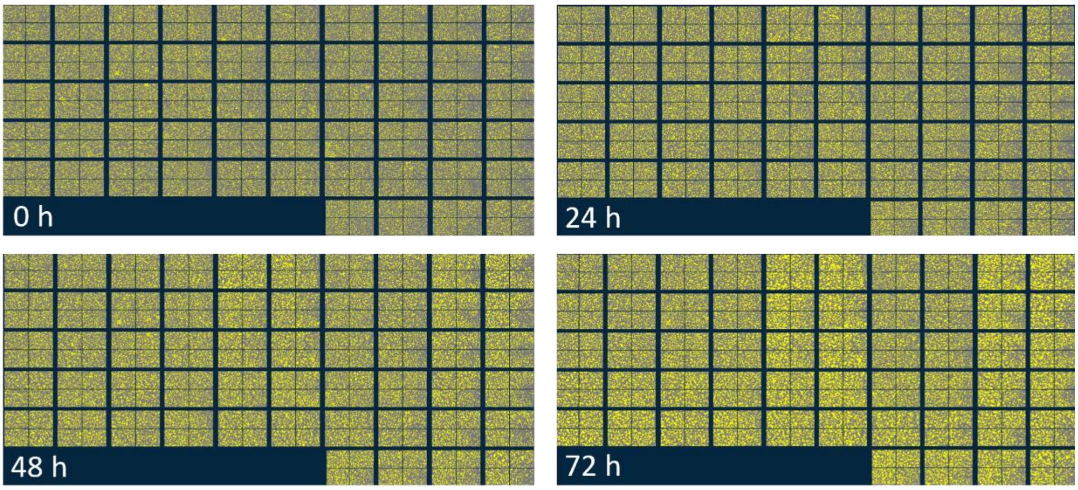

Figure S7. Representative images and plate map from proliferation assay at time points 0 h, 24 h, 48 h and 72 h imaged with Incucyte (Sartorius) live-cell imaging instrument. Phase mask colored with yellow.
